# Women in Control: Pioneering Diabetes Self-Management Medical Group Visits in the Virtual World

**DOI:** 10.4172/2167-0870.1000272

**Published:** 2016-06-28

**Authors:** S Mitchell, P Gardiner, G Weigel, M Rosal

**Affiliations:** 1 Department of Family Medicine, Boston Medical Center/Boston University School of Medicine, Boston, USA; 2 Department of Medicine, University of Massachusetts Medical School, USA

**Keywords:** Type 2 Diabetes, Virtual world, Medical group visits, Diabetes self-management, RCT

## Abstract

**Background:**

The current state of diabetes self-management (DSM) education and support for diabetic patients is inadequate, especially for minority women who experience disproportionately high rates of diabetes mellitus (DM) in the US. While DSM education and support enables individuals with diabetes to make positive lifestyle choices and achieve clinical goals, this type of support is difficult to deliver in medical practice settings. Virtual reality can assist DM patients and their clinical teams by providing effective educational tools in an engaging, learner-centered environment that fosters self-efficacy and skill proficiency.

**Methods:**

Our prior research demonstrated that virtual worlds are suitable for supporting DSM education. Building upon this success, we are now investigating whether DSM virtual world medical group visits lead to similarly effective health and educational outcomes compared to face-to-face medical group visits. Currently in year one of a five year randomized controlled trial, we aim to compare the effectiveness of a virtual world DSM medical group visit format versus a face-to-face DSM medical group visit format to increase physical activity and improve glucose control (HbA1c) among Black/African American and Hispanic women with uncontrolled DM. We will also conduct a qualitative study of participant engagement with the virtual world platform to characterize learners’ interactions with the technology and assess its correlation with DSM behaviors and diabetes control.

**Discussion:**

Novel methods to promote diabetes self-management are critically needed, and the use of virtual world technology to conduct medical group visits offers a unique approach to such issue. If successful, our intervention will increase access to culturally-sensitive diabetes care and improve patient engagement in online DSM learning, leading to higher uptake of DSM behaviors and better diabetes control. Importantly, the program can be easily expanded to other chronic disease areas and scaled for widespread use.

## Introduction

There are epidemic proportions of Type 2 diabetes mellitus (DM) in the US, with over 46.3% of individuals having T2DM or pre-diabetes as of 2014 [1]. Hispanic and non-Hispanic black women have the highest risk of developing DM in their lifetime as compared to non-Hispanic white women, and Hispanic, black and white males [2]. Hispanic and Black women also report low rates of physical activity, low consumption of fruits and vegetables, and low levels of diabetes self- efficacy, all significant risk factors for poor glycemic control and serious health complications.

Individuals who develop diabetes require diabetes self-management education and support to enable them to make good choices and achieve clinical goals; DSM education and group medical visits are more effective in improving diabetes outcomes than usual care, as they link lifestyle management with clinical care in peer-supported settings. This type of support and education, however, is difficult to deliver in medical practice settings [3]. With a shortage of healthcare providers and a growing prevalence of diabetes, there is great need for innovative approaches to DSM education and collaborative medical care to address disparities in DM outcomes.

Evidence suggests that computer based e-Health inventions that are personally relevant, contextually-situated, and culturally-tailored to the individual, are more likely to improve disparities in health outcomes and promote health behavior change, yet few studies focus on promoting DSM using the virtual world [4]. Virtual worlds are 3-D, computer based simulated environments, in which individuals create online manifestations of self through avatars and engage in experiences with other avatars on the platform. The use of virtual world technology for experiential learning is a new frontier for health behavior research and represents a novel opportunity for DSM education.

Preliminary evidence shows that virtual worlds influence “real world” behavior, as they are intrinsically designed for users to enact behavioral change, test new behaviors anonymously among peers, and restructure old habits [5–8]. As avatars, users explore environments, meet and socialize with others, and participate in group and individual activities. For some, the avatar becomes a purposeful projection or idealization of their own identify or an experiment with new identifies [9].

The virtual world offers a novel opportunity to improve health education by: (1) improving remote access to unique educational programs; (2) offering immersive personal learning environments; and (3) providing simulation-visualization exercises to enhance health behavior change. Virtual reality can assist DM patients and their clinical teams by providing effective educational tools in an engaging, learner-centered environment, fostering self-efficacy and skill proficiency.

Our recent pilot study, Women in Control 1.0, tested a virtual world DSM group education intervention with excellent results. The study was comprised of 89 African American women with uncontrolled DM, most of who were ≥ 40 years old (90%), from low-income households (82%) and had a high school education/equivalent (55%). Pilot data shows DSM group visits conducted in Second Life, a 3-D virtual lab, were not only feasible but as effective as face-to-face methods for improving DM disease control and diabetes self-efficacy, and showed significantly greater improvements in physical activity for virtual world participants [10].

Our current research, Women in Control 2.0, builds upon the virtual world DSM pilot program to include a medical consult consistent with the group medical visit model and a social ecological framework. While evidence shows the potential efficacy of Internet- based DSM education programs, to date, little work has focused on the use of virtual world technology for a group medical visit. To our knowledge, the Women in Control 2.0 intervention is the only virtual world program to include clinicians in a DSM group medical visit format.

## Methods/Design

### Objectives and hypotheses

Women in Control 2.0 is a randomized controlled trial aimed to test the comparative effectiveness of diabetes self-management medical group visits conducted in the virtual world versus a face-to-face format for Black/African-American and Hispanic/Latina women with uncontrolled diabetes. Our ongoing randomized controlled trial will assess the comparative effectiveness of the group visits in increasing physical activity and improving diabetes control (HbA1c) at six-month follow up. Based off our pilot data [10], we hypothesize the DSM medical group visits delivered in the virtual world will be non-inferior in increasing participant physical activity and improving HbA1c to visits conducted via face-to-face format. We will also conduct a qualitative, ethnographic study of participant engagement with the virtual world platform and assess its correlation with real-world DSM behaviors.

### Setting and participants

All study activities will be conducted in person, at Boston Medical Center, a large, academic teaching hospital, and online in the virtual world platform. Patients will be eligible if they are female, 18 years or older, self-identify as Black/African American or Hispanic/Latina, have been diagnosed with Type II Diabetes Mellitus (T2DM), had a HbA1c value above 8.0 at last reading, and are currently treated with diet, oral hypoglycemic agents and/or insulin. They must also have telephone access, can communicate in English or Spanish, can understand and provide informed consent and are functionally capable of meeting the activity goals in the goals. Participants will need their primary care provider’s approval before enrolling in the study. Participants will be excluded from the study if they have a history of diabetic ketoacidosis, are currently or planning pregnancy, are unable or unwilling to provide informed consent, have plans to leave the area the 6-months period of their involvement in the study, require intermittent glucocorticoid therapy within the last three months, have experienced an acute coronary event with the previous six months, have a medical condition that precludes adherence to study dietary recommendations or have a medical or serious psychiatric illness. Those with a diagnosis of depression or who take antidepressants are eligible.

### Study design

**Figure 1:**
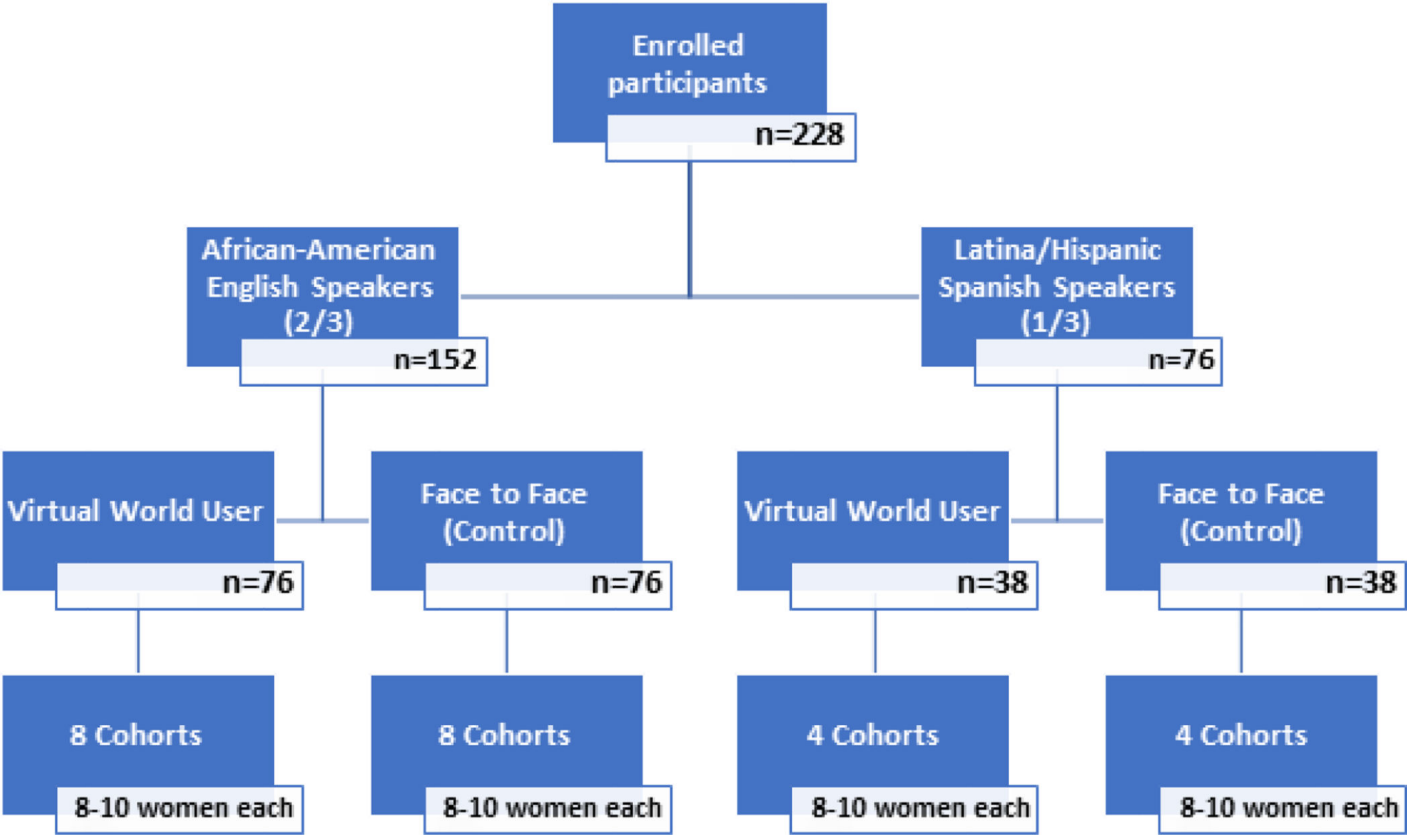
Schematic overview of trial design, broken down by language preference (English vs. Spanish) and randomization into intervention condition (Virtual world) and control condition (face to face). Each cohort for the medical group visits will consist of 8–10 women each.

A woman in Control 2.0 is a two-arm randomized control trial conducted at Boston Medical Center and in a virtual world platform. All study subjects will participate in 8-weeks of DSM medical group visits, either in the virtual world or face-to-face format. Participants will be stratified by preference of language (English or Spanish), and medical group visits will be conducted in one language or the other based on the cohort. After stratification by language, participants will be randomized into the experimental (virtual world users) and control conditions (face-to-face format) (Figure 1). Based off our hypotheses, we anticipate the virtual world format will be non-inferior, and perhaps superior, to the face to face format in improving glucose control and increasing physical activity among the medical group visit participants.

### Recruitment

We will recruit subjects from the Diabetes and Family Medicine clinics at BMC, and from the affiliated Boston HealthNet community health center network. PCPs will identify patients of theirs whom they believe would benefit from participating in the study. The PCP will send letters containing study information to the patient, and if interested, the patient will contact our research team by phone for initial screening. If the patient meets the inclusion criteria for the study based off the screening phone call and chart review by research staff, the patient will be scheduled for an in-person enrolment appointment. At this time, research staff will discuss with the patient the study design, requirements, scheduling of group visits and compensation, and the participant will give written informed consent. Baseline data measures will then be collected, and participants will receive a laptop, computer training and training on the virtual world platform.

### Intervention

At baseline, all study participants will be required to answer 9 validated questionnaires on various aspects of diabetes self- management and patient activation and engagement (Table 1). Participants will also have a baseline blood draw taken, to assess their HbA1c and cholesterol. For 1 week following baseline data collection, study subjects will wear an accelerometer, to track their physical activity and inactivity.

Post-enrolment and baseline data collection, medical group visits will commence weekly, either in the virtual world or face to face, based on the randomization assignment. Groups will consist of 8–10 women, and all study subjects will gather each week (~100 mins per week) with their group either online or in-person throughout the 8-week intervention. Led by a clinician and a peer-leader, participants will progress through an interactive and collaborative DSM curriculum with emphasis on promoting healthy food choices, physical exercise, stress reduction and mindfulness, while better understanding how diabetes and diabetes medications affect the body.

The Women in Control 2.0 DSM curriculum is based off the Women in Control 1.0 program, the CDC’s Power to Prevent curriculum [11] and the Integrative Medicine Group Visit curriculum [12], pioneered and developed at Boston Medical Center in the Department of Family Medicine. At each group session, participants will have an individual, private consultation with a clinician, lasting approximately 15 minutes. The virtual world participants will be able to log into the virtual platform at any time in between group visits, to interact with and explore the virtual world space.

After completion of the 8-weeks of medical group visits, the same outcome measures collected at baseline will be collected from participants at 8-weeks (post intervention) and at a 6-month follow up. Focus groups and key informant interviews from select individuals will be conducted post-intervention, to elicit their experiences with the DSM curriculum, virtual world platform (for virtual world users) and group medical visit.

### Outcomes

The primary outcomes of this study are changes in participants’ physical activity levels and measures of HbA1c. Physical activity will be measured in METs/hr; participants will wear an accelerometer for a one week period, at baseline, 8-weeks and 6-months. The data will be analyzed to determine the average change in total physical activity from baseline to 6 months of all participants by study arm (VW vs. face-to-face).

We will assess the average change in HbA1c (%) from baseline to 6 months of all participants by study arm from blood samples collected and analyzed at the BMC laboratory.

Secondary outcomes include patient activation, medication adherence, depression, clinical parameters (blood pressure, cholesterol, and weight/BMI), dietary patterns, stress levels, functional status, health literacy, social support, social network changes, quality of life and health service utilization [13–22].

We will also assess participant satisfaction with the curriculum, their confidence using computers, and self-reported adherence to DSM behaviors [23].

All study sessions will be audio and video recorded for both face-to- face and Second Life sessions. The primary, secondary and exploratory outcomes of this study are detailed in Table 1, along with their associated measurement tools and frequency of measurement.

### Sample size calculation

This study will consider the discrete difference in the average change in physical activity and HbA1c by study arm to be co-primary outcomes, as measured by calculating the different in physical activity levels and HbA1c levels from baseline to 6 month follow up for each participant, then determining the average change across all participants per study arm, and then between each arm. Due to the paucity of available data determining accelerometry cut-off points for populations with chronic disease [24], data obtained with the Women In Control 1.0 pilot study was used to estimate the sample size necessary to establish non-inferiority of SL to face-to-face in reducing HbA1c (%) and increasing total physical activity levels (MET/hr). If the improvements in the outcomes in the Second Life group exceed those in the face-to-face group, we will assess the superiority of the intervention condition over the control. The results of the pilot indicate that the SL group and face-to-face group had roughly similar discrete reductions in HbA1c on average from baseline to 6 months (−0.46 and −0.31, respectively, with a common standard deviation of 2). Assuming a non-inferiority margin of 0.7, a pooled standard deviation of 2, alpha of 0.05, and targeting a power of 80%, we require 102 participants per study arm to show non-inferiority of SL to face-to-face to account for a clinically meaningful decrease in HbA1c. Total physical activity, measured in MET/hr, increased significantly more from baseline to 6 months for the SL group when compared to the face-to-face group (6.70 vs. −9.20, respectively), with a pooled standard deviation of 35.We will assume a non-inferiority margin of 12, and under the assumption of a pooled standard deviation of 35, alpha of 0.05, and target power of 80%, we will require 106 participants per group, for a total of 212. Considering that the dropout rate for the pilot was low (5%), we expect the dropout rate for this study not to exceed 7%. With this consideration, we will enrol and randomize 228 participants, and expect to retain 212.

### Randomization

After stratification by language (English vs. Spanish), we will use one-to-one block randomization (alternating blocks of 6 and 8) into the intervention and control condition. Investigators are blinded to the randomization process, but the assignment will be revealed to the participant and the investigators thereafter in order to conduct the medical group visits.

### Data collection and management

Research data will be stored using Research Electronic Data Capture (REDCap), which is accessible online via a secure web portal. Participants will be assigned a study ID which will be used on all forms and records collected. Master lists linking participant information to their ID number will be password protected and only accessible by the PI and other investigators. Necessary hard copy records will be kept locked.

### Statistical analysis

Quantitative analysis will be conducted using SAS 9.3 and Microsoft Excel 2013. Significance will be determined at a 0.05-signifncance level for all tests. Analysis results will be compared for both primary outcomes utilizing non-inferiority margins of 0.7% for HbA1c and 12 METs/hr for total physical activity. All qualitative analysis of focus group and key informant interviews will utilize NVivo11 qualitative research software, and analysis will be conducted using grounded theory and constant comparative analysis.

### Ethical considerations

The Boston University Medical Center Institutional Review Board has approved this study protocol. All recruitment and study procedures will strictly adhere to HIPPA regulations. The Data and Safety Monitoring Board for this project will meet twice a year over the study period to review safety information including any adverse events possibly related to the interventions. All participants will give written informed consent, in their native language (English or Spanish), and all study activities for the individual will be conducted in that preferred language.

## Discussion

Women in Control 2.0 is a randomized controlled trial aimed to test the comparative effectiveness of diabetes self-management medical group visits conducted in the virtual world versus a face-to-face format for groups of Black/African-American and Hispanic/Latina women with uncontrolled diabetes. This research is necessary given the current inadequacy of diabetes self-management education and support for diabetic patients, especially for minority populations. Novel methods to promote diabetes self-management are critically needed, and the use of virtual world technology to conduct medical group visits offers a unique approach to such issue.

Although research in the use of virtual worlds for patient education and engagement is limited [25,26], we anticipate virtual world learning may offer participants several benefits over the face-to-face format. One of the most widely acknowledged benefits of the virtual world is the ease of distance learning. The virtual world allows participants to engage in learning from convenient locations, eliminating potential challenges due to transportation and mobility due to illness burden, and interact with a wide range of individuals who are not confined by geographic proximity.

Beyond distance learning, the virtual world offers novel approaches to behavioral change and experimentation with self-identity. In the virtual world, the participant customizes the appearance of the avatar as desired, which may or may not reflect their actual real-life appearance. This gives them the freedom to present themselves in more malleable fashion than in the physical world. Through actively controlling the avatars appearance, actions and speech, the participants is forced to be deliberate and intentional with their actions, which may lead to a heightened awareness of the connection between their intentions, actions and consequences through embodied learning [27]. Instead of learning in the abstract, as in a traditional classroom or support group, the avatar learns through experience in the virtual world while collaborating with other avatars, which then may translate to real life actions and behavioral change [28,29]. As learners experiment with their avatars, they are free to test out new behaviors, and even fail while doing so, without the real-life consequences normally attached to such actions [9,30]. In this fashion, participants can experience the negative and positive consequences of lifestyle modifications and behaviors, before making these changes in real life. This safe environment to learn, experiment, explore and collaborate with others is crucial when trying to enact behavioral change, and this type of applied learning in a personalized context has been associated with successful behavioral change and improved self- efficacy [31]. For these reasons, we anticipate the unique affordances of the virtual world will aid our virtual world participants greatly in their DSM efforts.

Ultimately, we aim to offer a tailored, individualized simulation-based learning experience that links DSM education with patients’ clinical care, daily lifestyle, peer support, home and virtual world community. If successful, this program will increase access to culturally-sensitive diabetes care and improve patient engagement in online DSM learning, leading to higher uptake of DSM behaviors and better diabetes control. Importantly, the program can be easily expanded to other chronic disease areas and scaled for widespread use.

## Figures and Tables

**Table 1:** Women in Control 2.0 Study Outcomes and Measures by Specific Aim.

Specific Aim	Outcome	Instrument	Timeline
(1) RCT of DSM Training Using Virtual World vs. Face to Face Format to Increase Physical Activity Levels			
Primary Outcome	Change in Physical Activity Level (METs/hr)	Change in accelerometer readings	Baseline, Post- interventio n & 6 months follow up
(2) RCT of DSM Training Using Virtual World vs. Face to Face Format to Reduce HbAlc Levels			
Primary Outcome	Change in disease control	Change in HbAlc from BMC laboratory blood testing results	
	Change in patient activation	Change in PAM 13 score	
	Medication Adherence	Prescription fulfilment ratio per 12b2 database claims data analysis	
	Depression	Patient Health Questionnaire (PHQ-8)	
Secondary Outcomes	Improvement in cholesterol, hypertension and body weight	Serum LDL/HDL, BP, BMI from BMC laboratory results, BP readings from home blood pressure kits, weight/ height measurements	Baseline, Post- interventio n & 6 months follow up
	Health Related Quality of Life	Q-LES-Q Screening Survey	
	Dietary Habits	Multiple measures of 24-hr dietary recall	
	Functional Status	Sheehan Disability Scale	
	Stress	Perceived Stress Scale (PSS-10) Survey	
	Social Support	MOS Social Support Survey	
	Health service utilization	Self-report & chart review of hospitalizations, PCP & specialist visits	
(3) Qualitative Ethnographic Study of Participant Behaviors in Virtual Environment			
(Virtual World [Second Life] Participants ONLY)			
	Measure of Positive Technological Development to assess influence of technology on learning and skill development	Positive Technological Development Questionnaire (PTDQ)	Baseline, Post- interventio n & 6 months follow up
Primary Outcomes	Characterize participants’ virtual world resource use, user patterns and peer interactions in Second Life	Ethnographic study of both learning environments from researcher’s field notes and session audio recordings	
	Characterize Patients’ perceived DSM educational experience	Key informant interviews with participants in virtual world and responses to PTDQ questionnaire	Performed during 8- week session blocks
	Characterize patient- clinician interactions in Second Life group visits	Ethnographic study of Second Life clinician consultations and key informant interviews	
Exploratory Outcomes	Changes in Social Network Metrics	Social Network Diagnostic Tool	Baseline, Post- interventio n & 6 months follow up
	Use of social media	Self-report tracking of logins for contracts with group peers via survey	Performed during 8- week session blocks

## Abbreviations

DM: Diabetes Mellitus
T2DM: Type 2 Diabetes Mellitus
DSM: Diabetes Self-Management
